# Expression and regulation of neurotrophins in the nondegenerate and degenerate human intervertebral disc

**DOI:** 10.1186/ar2487

**Published:** 2008-08-27

**Authors:** Devina Purmessur, Anthony J Freemont, Judith A Hoyland

**Affiliations:** 1 Tissue, Injury and Repair, School of Clinical and Laboratory Sciences, Faculty of Medical and Human Sciences, Stopford Building, Oxford Road, The University of Manchester, Manchester, M13 9PT, UK

## Abstract

**Introduction:**

The neurotrophins nerve growth factor (NGF) and brain-derived neurotrophic factor (BDNF) have been identified in the human intervertebral disc (IVD) and have been implicated in the mechanisms associated with nerve ingrowth and nociception in degeneration of the IVD. The aim of the current study was to investigate an association between neurotrophin expression in the IVD and the severity of disc degeneration, including the effect of disc-related proinflammatory cytokines on neurotrophin and neuropeptide expression in cells derived from the human IVD.

**Methods:**

Immunohistochemical analysis was performed to examine the expression of NGF, BDNF and their high-affinity receptors Trk-A and Trk-B in human IVD samples, divided into three categories: non-degenerate, moderate degeneration and severe degeneration. In order to study the effect of disc-related cytokines on neurotrophin/neuropeptide gene expression, nucleus pulposus cells derived from non-degenerate and degenerate IVD samples were seeded in alginate and were stimulated with either IL-1β or TNFα for 48 hours. RNA was extracted, cDNA was synthesised and quantitative real-time PCR was performed to examine the expression of NGF, BDNF and substance P.

**Results:**

Immunohistochemistry showed expression of NGF and BDNF in the native chondrocyte-like cells in all regions of the IVD and in all grades of degeneration. Interestingly only BDNF significantly increased with the severity of degeneration (*P *< 0.05). Similar expression was observed for Trk-A and Trk-B, although no association with disease severity was demonstrated. In cultured human nucleus pulposus cells, stimulation with IL-1β led to significant increases in NGF and BDNF gene expression (*P *< 0.05). Treatment with TNFα was associated with an upregulation of substance P expression only.

**Conclusion:**

Our findings show that both the annulus fibrosus and nucleus pulposus cells of the IVD express the neurotrophins NGF and BDNF, factors that may influence and enhance innervation and pain in the degenerate IVD. Expression of Trk-A and Trk-B by cells of the nondegenerate and degenerate IVD suggests an autocrine role for neurotrophins in regulation of disc cell biology. Furthermore, modulation of neurotrophin expression by IL-1β and modulation of substance P expression by TNFα, coupled with their increased expression in the degenerate IVD, highlights novel roles for these cytokines in regulating nerve ingrowth in the degenerate IVD and associated back pain.

## Introduction

Low back pain is a widespread and incapacitating disorder of significant social and economic importance [1]. Degeneration of the intervertebral disc (IVD) has been implicated in the pathogenesis of chronic low back pain [2], and is characterised by increased degradation of the extracellular matrix coupled with an ingrowth of blood vessels and nerves [3,4] into the normally avascular and aneural tissue. Indeed, we have shown an association between the painful degenerate IVD and nerve ingrowth [5]. The mechanisms underlying innervation into the degenerate human IVD, however, are largely unknown. Studies by Johnson and colleagues have demonstrated that the extracellular matrix component aggrecan, derived from the healthy IVD, has an inhibitory effect on neurite outgrowth [6]. Likewise, such an inhibitory effect has also been demonstrated in an ovine annular model of disc degeneration in which depletion of the proteoglycan content was associated with an increased nerve ingrowth into the IVD [7].

The normal healthy IVD is largely aneural. Anatomical studies by Jackson and colleagues and by Bogduk and colleagues have demonstrated innervation of only the superficial outer layers of the annulus fibrosus, with the central core of the IVD completely lacking nerves [8,9]. This is supported by the work of Fagan and colleagues, who have demonstrated innervation of both the perianular region of the IVD as well as the central region of endplate above the nucleus pulposus (NP) [10]. Within the degenerate IVD, however, nerves have been shown to penetrate both the inner annulus fibrosus (IAF) and the NP. Importantly, these innervating fibres have been shown to express both the neural growth-associated marker GAP43 and the pain-associated neuropeptide substance P [5].

Additional studies investigating innervation in the degenerate IVD have also demonstrated expression of Trk-A, the high-affinity receptor for nerve growth factor (NGF), on ingrowing nerves – indicating that such nerves are NGF sensitive [4]. This is supported by Aoki and colleagues, who have identified NGF-responsive nerve fibres as the predominant neuronal subtype innervating the peripheral regions of the healthy annulus fibrosus [11]. Studies by Ohtori and colleagues have also demonstrated that a number of the neurons innervating the rat IVD were immunoreactive for brain-derived neurotrophic factor (BDNF), a modulator of pain-associated processes in the dorsal horn of the spinal cord [12].

Neurotrophins are growth and survival factors, associated mainly with neuronal development, function and nociception [13]. They are located primarily within the central and peripheral nervous systems, and they consist of a family of four principal members: NGF, BDNF, neurotrophin 3 and neurotrophin 4/5. Both NGF and BDNF have been identified in non-neural cells of healthy and diseased connective tissue such as human cartilage and bone, and, interestingly, recent studies have highlighted a possible role for neurotrophins in the IVD [14-17]. For example, Johnson and colleagues demonstrated stimulation of neurite outgrowth by soluble mediators produced by degenerate disc cells, mediators they hypothesised to be neurotrophic factors [18]. Additionally, NGF has been identified in association with the blood vessels growing into the degenerate IVD [4], and also in the rounded chondrocyte cells of the annulus fibrosus [16]. More recently, Abe and colleagues have confirmed this and have also shown both expression and synthesis of NGF in NP cells isolated from the human IVD [19].

As well as NGF expression, microarray analysis of human disc cells subjected to hyperosmotic stimuli has identified expression and upregulation of BDNF [20]. Additionally, in a recent study Gruber and colleagues showed expression of BDNF in human IVD cells, with BDNF gene expression in annulus fibrosus cells positively correlating with increasing degeneration [21]. Whether both NGF and BDNF are upregulated in the degenerate IVD, and as such play a key role in innervation and the pathogenesis of low back pain, however, remains to be determined.

The neurotrophins NGF and BDNF function principally through their high-affinity receptors Trk-A and Trk-B. Indeed, both receptors have been identified in cells of the musculoskeletal system, and more recent studies have demonstrated Trk-A and Trk-B expression in human disc cells [14-17,19,21]. Studies investigating a role for neurotrophins in the pathogenesis of osteoarthritis have shown upregulation of both NGF and Trk-A in osteoarthritic chondrocytes [15]. Nevertheless, expression of Trk-A and Trk-B by human disc cells in terms of disease pathology has yet to be fully examined.

A role for the proinflammatory cytokines TNFα and IL-1β in innervation of the IVD [22] and in degeneration of the IVD has been demonstrated by a number of authors [23-25]. Additionally these cytokines have been shown to regulate neurotrophin expression in non-neuronal cells. Studies by Kemi and colleagues have demonstrated upregulation of NGF and BDNF in bronchial smooth muscle cells after treatment with IL-1 [26]. Similarly, in synovial fibroblasts isolated from patients with osteoarthritis, both IL-1β and TNFα were shown to increase NGF synthesis and release [27]. Evidence to support a role for IL-1β and TNFα in the modulation of neurotrophin expression in the IVD comes from studies by Abe and colleagues, who demonstrated an increase in NGF gene expression and protein secretion after NP cells (cultured in monolayer) were treated with IL-1β and TNFα [19].

Work to date therefore supports a role for neurotrophins in the biology of innervation in the IVD, the function of which may be regulated by proinflammatory cytokines known to be upregulated during IVD degeneration [23-25]. Based on this knowledge we hypothesised that expression of the neurotrophins NGF and BDNF increases with disc degeneration and that they are regulated by IL-1β, which also increases with the severity of degeneration. Using immunohistochemistry we have therefore investigated NGF and BDNF expression, including expression of the receptors Trk-A and Trk-B, in non-degenerate, moderately degenerate and severely degenerate human IVD tissue, and we have examined the effects of IL-1β and TNFα on neurotrophin expression in non-degenerate and degenerate disc cells (seeded in a three-dimensional matrix).

## Materials and methods

### NGF, BDNF, Trk-A and Trk-B protein expression in the nondegenerate and degenerate human IVD

Human IVD tissue was obtained at surgery/postmortem, and ethical consent was obtained from the local ethical committees together with informed consent from the patient or relatives. IVD tissue samples were fixed overnight in 4% paraformaldehyde/PBS at 4°C, processed to paraffin wax and 5 μm sections prepared.

For immunohistochemical analysis, a total of 15 patient samples were used (age range 37 to 75 years): non-degenerate IVDs (grades 1 to 4), moderately degenerate IVDs (grades 5 to 8) and severely degenerate IVDs (grades 9 to 12), with five patient samples per category. Grading of IVD tissue was carried out as described by Sive and colleagues [28].

### Immunohistochemical procedure

The immunohistochemical protocol was as previously published [20]. Briefly, following blocking of endogenous peroxidase and antigen retrieval with chymotrypsin (1% w/v) at 37°C for 20 minutes, sections were incubated overnight at 4°C with either 15 μg/ml primary goat anti-NGF, 15 μg/ml primary mouse anti-BDNF, 10 μg/ml primary goat anti-Trk-A or 8 μg/ml primary mouse anti-Trk-B antibodies (R&D Systems, Abingdon, UK). Negative controls were used in which respective goat and mouse IgGs (R&D Systems) replaced the primary antibody. After washing, sections were incubated with biotinylated donkey anti-goat and goat anti-mouse secondary antibodies (Santa Cruz, CA, USA) at dilutions of 1/300 and 1/400, respectively, for 30 minutes at room temperature. Disclosure of secondary antibody binding was by the streptavidin–biotin complex (Dako, Cambridgeshire, UK) technique with 3,3'-diaminobenzidine tetrahydrochloride solution (Sigma, Poole, UK). Sections were counterstained with Mayers Haematoxylin (Raymond. A. Lamb, East Sussex, UK), were dehydrated and were mounted in XAM (BDH, Liverpool, UK).

### Analysis of immunohistochemical data

For each tissue section, the different regions of the disc – NP, IAF and outer annulus fibrosus (OAF) – were first identified based on the cell morphology and the structure of the extracellular matrix. Up to a total of 400 cells were counted in each region of the IVD, and the number of positively stained cells was expressed as a percentage of the total number.

### Statistical analysis

All data were tested for normality using the Shapiro–Wilke W method of analysis. As the data did not follow a normal distribution, a two-sided Mann–Whitney U test was performed to determine significance between the percentage of positive cells in the non-degenerate, moderate and severely degenerate disc samples. A Wilcoxin test was used to identify significance between the different regions of the IVD (NP, IAF and OAF). *P *< 0.05 was considered significant.

### Effect of proinflammatory cytokines on neurotrophin and neuropeptide gene expression in nondegenerate and degenerate nucleus pulposus cells

Primary NP cells were isolated from IVD samples obtained at surgery/postmortem, and ethical consent was obtained from the local ethical committees together with informed consent from the patient or relatives. NP cells were isolated from three non-degenerate IVDs (grades 1 to 2) of patients aged 37 to 61 years, and from three degenerate IVDs (grades 7 to 10) of patients aged 37 to 79 years.

### Nucleus pulposus cell extraction

For cell extraction, NP tissue samples were minced and digested with 2 U/ml protease in DMEM and Ham's F-12 media for 30 minutes at 37°C. To isolate NP cells, tissue samples were treated with 0.4 mg/ml collagenase type 1 (Gibco, Paisley, UK) for 4 hours in a shaking water bath at 37°C. The resulting cell suspension was passed through a 40 μm nylon cell strainer (BD Falcon, BD Biosciences, Oxford, UK) and spun at 300 × *g *for 10 minutes. The cell pellet was washed twice with DMEM and Ham's F-12. Cells were seeded in vented culture flasks (BD Falcon) and were fed with DMEM (glucose and pyruvate) supplemented with 10% v/v heat-inactivated FCS (Gibco), 100 U/ml penicillin, 100 μg/ml streptomycin, 250 ng amphotericin, 2 mM glutamine and 50 μg/ml ascorbate (NP 10% v/v serum-supplemented media). Cells were incubated at 37°C, 5% carbon dioxide and were fed with media every 3 to 4 days.

### Nucleus pulposus cell culture and treatment with proinflammatory cytokines

Non-degenerate and degenerate NP cells were seeded in alginate to maintain their native phenotype. Cells expanded in monolayer culture (passage 4 or less) were trypsinised and resuspended in 1.2% w/v low-viscosity alginate (Sigma) in 0.15 M NaCl to give a cell density of 4 million cells/ml. The alginate/cell suspension was drawn into a 5 ml syringe through a 21-gauge needle and drops of alginate/cell suspension were suspended in 102 mM CaCl_2_. Once formed, the alginate beads were placed on a rolling platform for 10 minutes at room temperature. This was followed by three 10-minute washes in 0.15 M NaCl followed by one final wash in NP 10% v/v serum-supplemented media. Beads were resuspended in NP 10% v/v serum-supplemented media and were plated into 12-well culture plates with approximately 10 to 15 beads per well. The plates were incubated for 14 days at 37°C, 5% CO_2 _and the media was changed every 3 days.

For cytokine treatment, six alginate beads were added in triplicate to wells of a 12-well plate with 2 ml media and were then treated with 10 ng/ml IL-1β (AMS Biotechnology Ltd, Abingdon, UK) or with 10 ng/ml TNFα (AMS Biotechnology Ltd) in NP 10% v/v serum-supplemented media for 48-hour incubation at 37°C, 5% CO_2_.

### Quantitative real-time PCR for NGF, BDNF and substance P expression

Post cytokine treatment RNA was extracted from NP cells, cDNA synthesised and quantitative real-time PCR carried out. Briefly, alginate beads were removed and 1 ml dissolving buffer (55 mM sodium citrate, 30 mM ethylenediamine tetraacetic acid and 0.15 M NaCl) was added to each NP sample and incubated at room temperature for 10 minutes. The solution was then centrifuged and the cell pellet lysed with 1 ml TRIzol^® ^reagent. Superscript II enzyme was used to reverse-transcribe 500 ng RNA into cDNA in a standard 20 μl reaction (Invitrogen, Paisley, UK).

Quantitative real-time PCR was carried out using the predesigned FAM MGB combined primer and the probe sets termed assays on demand for NGF, BDNF and substance P from Applied Biosystems (Warrington, UK): NGF, Hs00171458_m1; BDNF, Hs00542425_s1; substance P, Hs00243225_m1. For each 25 μl reaction, 2.5 μl cDNA was added to 12.5 μl Taqman Universal PCR Master Mix (Applied Biosystems), 1.25 μl assays on demand and 8.75 μl RNase-free molecular-grade water.

All cDNA samples and reactions were carried out in duplicate in ABI Prism 96-well plates (Applied Biosystems)). The quantitative real-time PCR reactions were performed using an ABI Prism 7000 detection system (Applied Biosystems). The procedure involved denaturation, Taq activation for 10 minutes at 95°C, amplification with 40 PCR cycles of denaturation at 95°C for 15 seconds, followed by annealing and extension at 60°C for 1 minute. The Ct values generated were analysed as described both by Le Maitre and colleagues and by Livak and Schmittgen [23,29].

### Statistical analysis of quantitative real-time PCR data

To perform statistical analysis of all quantitative real-time PCR data, the Ct values from duplicates were averaged and the 2^-ΔCt ^value calculated from the Ct difference. These values were then grouped together into untreated, IL-1β-treated or TNFα-treated nondegenerate and degenerate NP samples, and the statistical significance was determined between untreated and corresponding cytokine treatments. All data were tested for normality using the Shapiro–Wilke W method of analysis. As the data did not follow a normal distribution, a two-sided Mann–Whitney U test was carried out to determine the significance between untreated and treated NP cell samples and also between non-degenerate and degenerate NP cell samples. *P *< 0.05 was considered significant.

## Results

### NGF and BDNF protein expression in the non-degenerate and degenerate human IVD

**Figure 1:**
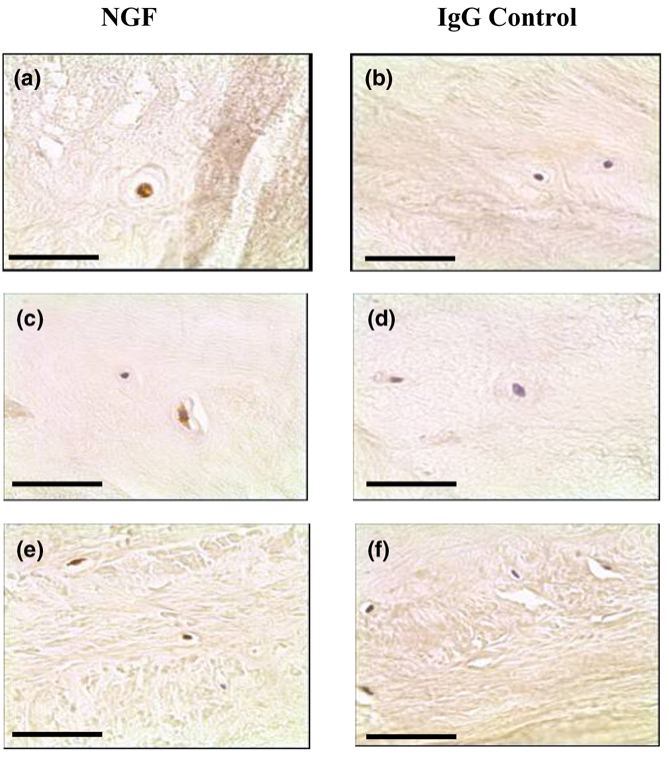
Nerve growth factor expression in human intervertebral disc tissue. Nerve growth factor (NGF) protein expression was identified in **(a)**, **(c) **the chondrocyte-like cells of human nucleus pulposus and inner annulus fibrosus tissue, and **(e) **the fibroblast-like cells of the outer annulus fibrosus. **(b)**, **(d)**, **(f) **IgG controls were negative. Scale bar = 50 μm.

**Figure 2:**
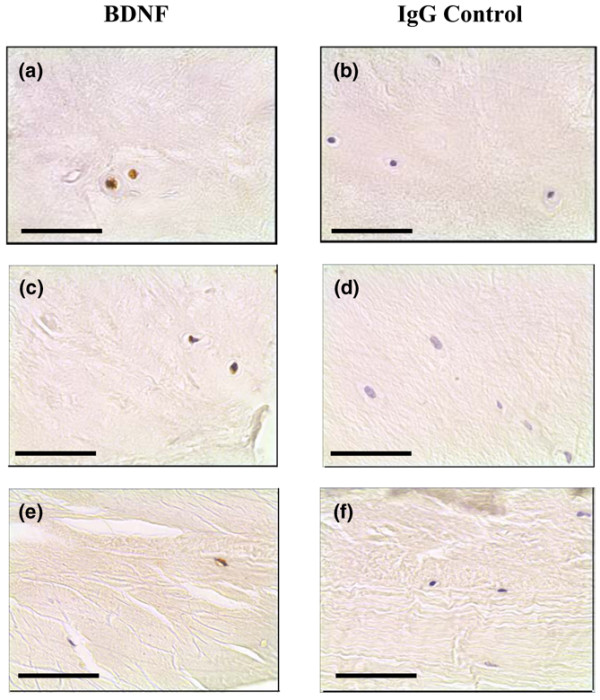
Brain-derived neurotrophic factor expression in human intervertebral disc tissue. Brain-derived neurotrophic factor (BDNF) protein expression was identified in **(a)**, **(c) **the chondrocyte-like cells of human nucleus pulposus and inner annulus fibrosus tissue, and **(e) **the fibroblast-like cells of the outer annulus fibrosus. **(b)**, **(d)**, **(f) **IgG controls were negative. Scale bar = 50 μm.

Immunopositive staining was observed for both neurotrophins. Cellular staining for NGF and BDNF was identified in the native chondrocyte-like cells of the NP and IAF tissue and in the fibroblast-like cells of the OAF (Figures 1a to 1f and 2a to 2f). The cell bodies of pyramidal neurons in human brain control tissue were also positive. Immunopositivity was observed in all regions of the IVD and was present in both non-degenerate and degenerate states.

**Figure 3:**
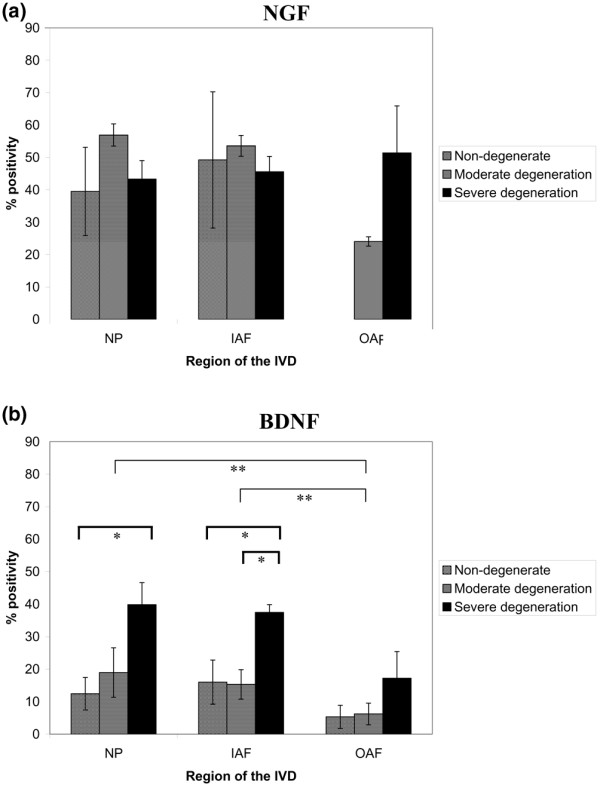
Neurotrophin immunopositivity in the nondegenerate and degenerate intervertebral disc. Histograms illustrating the percentage of **(a) **positive nerve growth factor (NGF) cells and **(b) **brain-derived neurotrophic factor (BDNF) immunopositive cells in the nucleus pulposus (NP), inner annulus fibrosus (IAF) and outer annulus fibrosus (OAF) regions of nondegenerate, moderately degenerate and severely degenerate intervertebral disc (IVD) tissue. *Significance between disease states, **significance between regions of the IVD (*P *< 0.05). Data presented as the mean ± standard error of the mean.

For NGF, expression was greater in NP and IAF tissue of moderate degenerate samples (grades 5 to 8) when compared with both non-degenerate and severely degenerate samples, although these differences were not significant (Figure 3a). For the NP, 39.5%, 56.9% and 43.3% of cells were positive in non-degenerate, moderately degenerate and severely degenerate IVD tissue, respectively. For the IAF, 49.2%, 53.6% and 45.6% immunopositivity was identified in the non-degenerate and two degenerate states, respectively. For the OAF, 24.1% and 51.4% of cells were positive in moderately and severely degenerate IVD tissue, respectively. No significant differences were observed in cellular immunopositivity between the NP, IAF and OAF (*P *> 0.05).

An increase in BDNF protein expression was demonstrated with an increase in disease severity for all regions of the IVD (Figure 3b). For the NP, significance was observed between non-degenerate and severely degenerate tissue only: 12.5% compared with 39.8% (*P *< 0.05). For the IAF, however, significant differences were demonstrated for both the non-degenerate and moderately degenerate IVD compared with the severely degenerate IVD; 16.0% and 15.3% compared with 37.4% (*P *< 0.05). No significant differences were observed between any of the disease states in the OAF (*P *> 0.05). Levels of immunopositivity in the NP and IAF differed significantly from that of the OAF (*P *< 0.05).

### Trk-A and Trk-B protein expression in the non-degenerate and degenerate human IVD

**Figure 4:**
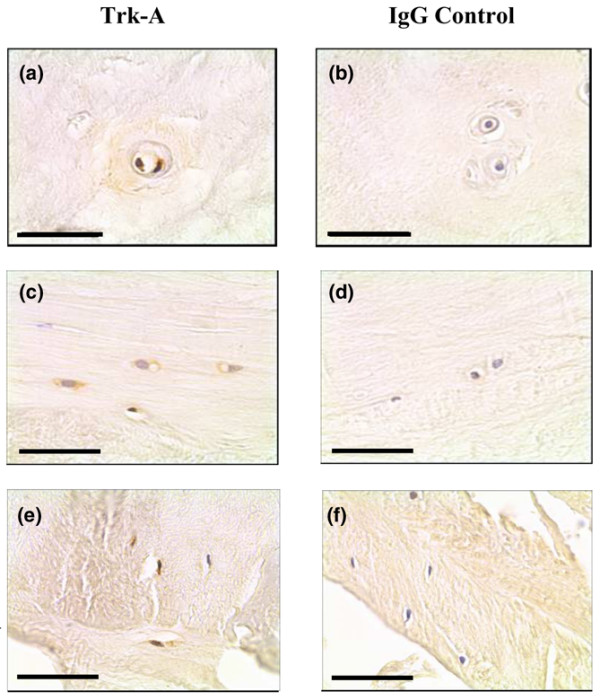
Trk-A expression in the human intervertebral disc tissue. Trk-A protein expression was identified in **(a)**, **(c) **the chondrocyte-like cells nucleus pulposus and inner annulus fibrosus tissue, and **(e) **the fibroblast-like cells of the outer annulus fibrosus. **(b)**, **(d)**, **(f) **IgG controls were negative. Scale bar = 50 μm.

**Figure 5:**
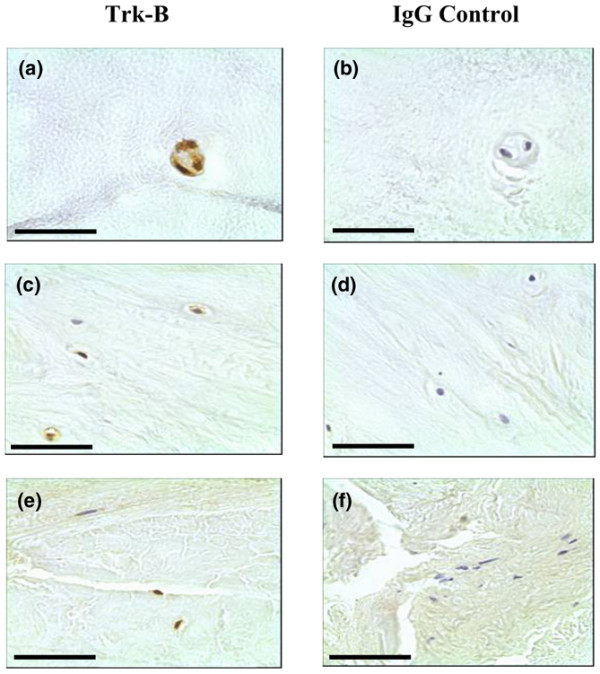
Trk-B expression in the human intervertebral disc tissue. Trk-A protein expression was identified in **(a)**, **(c) **the chondrocyte-like cells nucleus pulposus and inner annulus fibrosus tissue, and **(e) **the fibroblast-like cells of the outer annulus fibrosus. **(b)**, **(d)**, **(f) **IgG controls were negative. Scale bar = 50 μm.

Immunopositive staining for Trk-A and Trk-B was largely cellular in the chondrocyte-like cells of the NP and IAF, and also in the fibroblast-like cells of the OAF (Figures 4c,d and 5c,d). Positive staining in control brain tissue was localised to blood vessels and to pyramidal cells for Trk-A and Trk-B, respectively.

**Figure 6:**
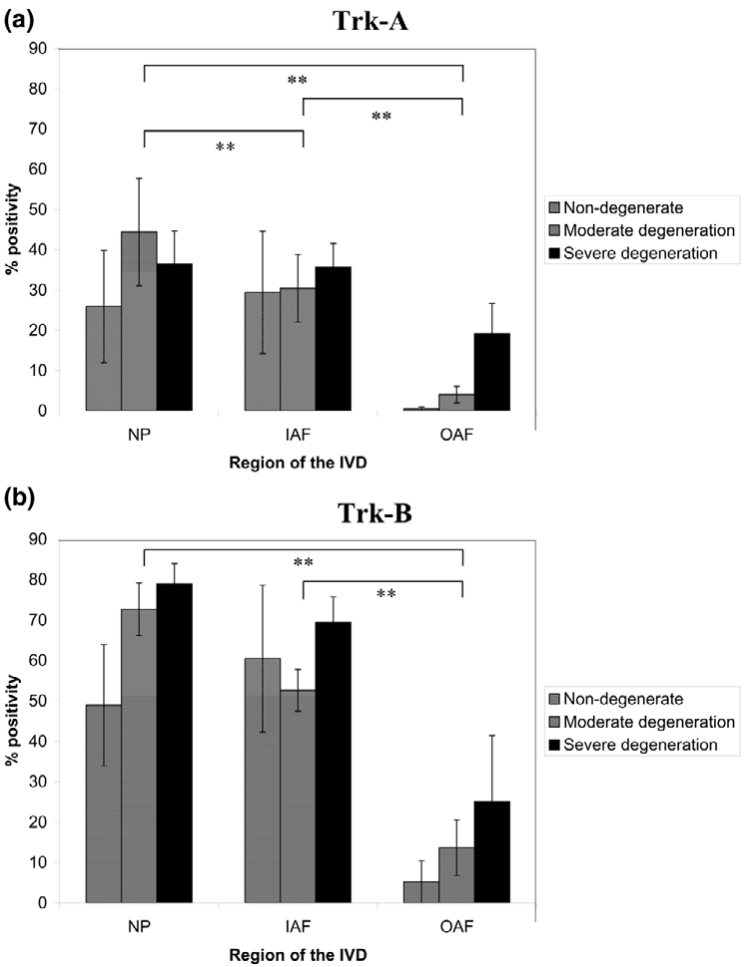
Neurotrophin receptor immunopositivity in the nondegenerate and degenerate intervertebral disc. Histograms illustrating the percentage of **(a) **positive Trk-A cells and **(b) **Trk-B immunopositive cells in the nucleus pulposus (NP), inner annulus fibrosus (IAF) and outer annulus fibrosus (OAF) regions of nondegenerate, moderately degenerate and severely degenerate intervertebral disc (IVD) tissue. **Significance between regions of the IVD (*P *< 0.05). Data presented as the mean ± standard error of the mean.

Expression was detected in the NP, IAF and OAF of non-degenerate, moderately degenerate and severely degenerate IVD tissue in all regions of the IVD (Figure 6). For Trk-A, no significant increase in immunopositivity with disease severity was observed (Figure 6a), although there were significant differences between levels of immunopositivity in the NP and IAF, and also in the NP and IAF compared with the OAF (*P *< 0.05). For Trk-B, increased numbers of immunopositive cells were observed in severe degeneration in all regions of the IVD, although this was not significant (Figure 6b). There were significant differences in immunopositivity between the OAF and both the NP and IAF (*P *< 0.05).

### Effect of proinflammatory cytokines on neurotrophin and neuropeptide gene expression in non-degenerate and degenerate nucleus pulposus cells

**Figure 7:**
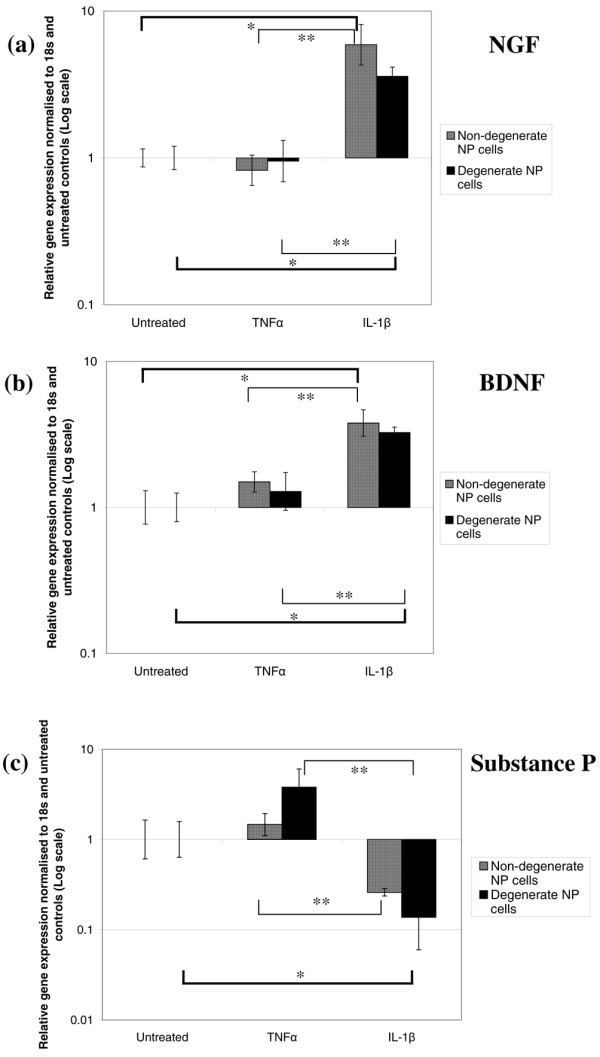
Effect of proinflammatory cytokines on neurotrophin and neuropeptide gene expression in nucleus pulposus cells. Histograms illustrating the relative gene expression of **(a) **nerve growth factor (NGF), **(b) **brain-derived neurotrophic factor (BDNF) and **(c) **substance P in nondegenerate and degenerate nucleus pulposus (NP) cells cultured with IL-1β and TNFα normalised to 18s and untreated controls. *Significance between IL-1β treatment and untreated control, **significance between IL-1β and TNFα treatments (*P *< 0.05). Data presented as the mean ± standard error of the mean.

To examine the effect of disc-related proinflammatory cytokines on neurotrophin and neuropeptide expression in the IVD, cells derived from non-degenerate and degenerate NP patient samples were treated with either IL-1β or TNFα. When these NP cells were treated with IL-1β for 48 hours, significant increases in the relative gene expression of NGF were observed (*P *< 0.05): increases of eightfold for non-degenerate NP cells and of 5.5-fold for degenerate NP cells were demonstrated compared with untreated controls (Figure 7a). TNFα had little effect on the relative gene expression of NGF in non-degenerate and degenerate NP cells. The effects demonstrated by IL-1β and TNFα on NP samples were significantly different from one another (*P *< 0.05), with IL-1β inducing greater NGF gene expression.

When observing the effects on BDNF gene expression, treatment with the proinflammatory cytokine IL-1β also led to significant increases – with changes of 5.5-fold in non-degenerate NP cells and fivefold in degenerate NP cells compared with untreated control cells (*P *< 0.05) (Figure 7b). Increases in BDNF gene expression of twofold and 1.5-fold were demonstrated after treatment with TNFα (*P *> 0.05). Significant differences were again observed between IL-1β and TNFα treatment for the NP samples tested (*P *< 0.05).

For the neuropeptide substance P, treatment with IL-1β led to a decrease in relative gene expression – with changes of sixfold in non-degenerate NP cells and eightfold in degenerate NP cells compared with untreated controls (Figure 7c). This change was significant for degenerate NP cells only (*P *< 0.05). Addition of TNFα had the opposite effect on substance P gene expression: increases of twofold in non-degenerate NP cells and sixfold in degenerate NP cells were observed compared with untreated controls (*P *> 0.05). Significance was demonstrated between IL-1β and TNFα treatments for both NP samples (*P *< 0.05)

## Discussion

A role for the neurotrophin family of growth factors, in particular NGF and BDNF, has been proposed in the IVD. There are no studies to date, however, which have examined an association between the expression of these two neurotrophins and the severity of disc degeneration. In the present study, an association between BDNF protein expression and disease severity was demonstrated – a greater number of positive disc cells was noted in all regions of severely degenerate samples (grades 9 to 12), which was significant for cells in the NP and IAF. For NGF, although a greater number of immunopositive cells were observed in the NP and IAF of disc samples of moderate degeneration and in the OAF of severely degenerate samples, these differences were not significant. These observations suggest that BDNF may be the more important neurotrophin in IVD degeneration driving pain-related processes as well as neuronal survival and innervation.

Few studies have examined the function of neurotrophin expression in cells of non-neural origin. Studies by Johnson and colleagues support the concept that disc cells, in particular cells from the degenerate IVD, produce nerve-promoting factors with the ability to induce neurite outgrowth [18]. Similarly, our previous work demonstrating the presence of Trk-A-expressing nerves growing into the degenerate IVD corroborates a role for neurotrophic factors, principally NGF, in the mechanisms associated with nerve ingrowth into the painful IVD [4]. Additionally, the high levels of NGF expression in both the non-degenerate and degenerate IVD perhaps point towards a function for NGF unrelated to nerve ingrowth or survival. Indeed, studies by Uchiyama and colleagues have highlighted a role for NGF in adaptation of disc cells to their acidic and hyperosmotic microenvironment via regulation of acid-sensing ion channel 3 [30].

Our data show increased expression of BDNF with increasing severity of degeneration, which supports the recent findings of Gruber and colleagues [21]. This highlights a novel function for BDNF in possible innervation in the degenerate IVD, although further work elucidating its exact role is necessary and should include investigation of BDNF-responsive nerves within the degenerate IVD. Similarly a role for neurotrophic factors with regard to nociception in the painful IVD has yet to be elucidated.

Using a rat model of experimental IVD herniation, Onda and colleagues have highlighted a correlation between induction of NGF within the dorsal nerve root using surgical procedures and pain behaviour [31]. In a mouse model of skeletal pain and of bone healing, antibodies blocking NGF were shown to significantly reduce fracture pain-related behaviours [32]. The function of BDNF with regard to pain processes has focussed mainly on its role within the dorsal horn and central nervous system, with few studies examining effects peripherally. It is possible the neurotrophic factors expressed by IVD cells may have the ability to modulate each other's function. Studies by Zhao and colleagues suggest that NGF may regulate inflammatory pain in sensory nociceptive neurons in part through the expression of BDNF [33]. A relationship between NGF and BDNF within the IVD therefore requires futher investigation.

The present investigation also importantly highlights a possible function for neurotrophins and their receptors in disc cell biology. In non-degenerate, moderate and severely degenerate IVD tissue, the receptors Trk-A and Trk-B were expressed by the chondrocyte-like cells of the IVD, suggesting that neurotrophins in the IVD may exert autocrine effects on the disc cells themselves. Significant differences were observed between receptor expression in the different regions of the IVD: expression was significantly greater in the NP and IAF compared with the OAF. Some investigators have suggested that neurotrophin and receptor expression by articular chondrocytes may contribute to disease processes by influencing cell metabolism [15]. Work by Raucci and colleagues has demonstrated NGF to have an inhibitory effect on cell proliferation in chondrocytes [34], and similar effects have been observed in osteoblasts [35]. Neurotrophins may therefore play a role in matrix turnover and regulation of cell metabolism in the IVD.

The study undertaken here confirms a role for disc-related proinflammatory cytokines IL-1β and TNFα in the modulation of neurotrophin and neuropeptide expression in cells from the IVD seeded in a three-dimensional matrix. Treatment with IL-1β resulted in a significant increase in the relative gene expression of both NGF and BDNF in NP cells, with a greater increase in cells from non-degenerate IVD. This supports the work of Abe and colleagues, who demonstrated an increase in NGF protein and gene expression in NP cells seeded in monolayer and treated with IL-1β [19]. Indeed a number of studies have demonstrated regulation of NGF expression and NGF-associated pain processes by IL-1β [27,33]. As a consequence, IL-1β may be an important factor with regard to upregulation of neurotrophin expression and nerve ingrowth by disc cells in the initial stages of disease. IL-1β expression in the IVD has also been shown to increase with disease severity [23], so it is reasonable to hypothesise that, as well as functioning to enhance catabolic processes, increasing amounts of IL-1β may also serve to perpetuate the degenerate disease IVD phenotype in terms of processes associated with nerve ingrowth and pain.

Unlike the results obtained by Abe and colleagues, however, the studies described here showed TNFα to have little effect on neurotrophin gene expression [19]. This may be as a consequence of differences in experimental procedures, as we cultured NP cells in alginate – which maintains a phenotype more representative of that seen *in vivo *– whereas Abe and colleagues treated NP cells cultured in monolayer [19]. Work by Hattori and colleagues has demonstrated a synergistic effect between TNFα and IL-1β in the stimulation of NGF from fibroblasts [36], and therefore whether the effects of TNFα on neurotrophin expression in the IVD also requires the presence of IL-1β remains to be investigated.

It is possible to hypothesise that substance P may contribute to nociceptive processes within the degenerate IVD since expression of the pain-related neuropeptide and its receptor have been identified in both disc cells and nerves growing into the diseased IVD [5,37-39]. In the present study TNFα treatment resulted in an increase in substance P expression, and this was greater in degenerate NP cells. An association of TNFα with substance P expression and degeneration is supported by the work of Chubinskaya and colleagues, who have demonstrated downregulation of both genes in an anticatabolic disc model [40].

## Conclusion

The results overall suggest a dual function for neurotrophins within the non-degenerate and degenerate IVD. Expression of NGF and increased expression of BDNF with degeneration identifies a possible role for these factors in perpetuation of nerve ingrowth into the degenerate IVD. Expression of the high-affinity receptors by cells of the non-degenerate and degenerate IVD, however, implicates an autocrine role for neurotrophins in disc cell metabolism. Enhancement of neurotrophin expression by IL-1β and neuropeptide expression by TNFα coupled with the increased expression of these proinflammatory cytokines in the degenerate IVD identifies new roles for IL-1β and TNFα in regulating nerve ingrowth and associated chronic low back pain. IL-1β may act as a key modulator of neurotrophin expression in the IVD, yet TNFα may have an influential role in regulation of nociception through substance P.

## Abbreviations

BDNF = brain-derived neurotrophic factor; DMEM = Dulbecco's modified Eagle's media; FCS = foetal calf serum; IAF = inner annulus fibrosus; IL = interleukin; IVD = intervertebral disc; NGF = nerve growth factor; NP = nucleus pulposus; OAF = outer annulus fibrosus; PBS = phosphate-buffered saline; PCR = polymerase chain reaction; TNF = tumour necrosis factor.

## Competing interests

The authors declare that they have no competing interests.

## Authors' contributions

DP participated in the design of the study, performed the majority of the laboratory work and analysis, and drafted the manuscript. AJF helped to secure funding, participated in the design of the study and interpretation of data, and assisted in the preparation of the final manuscript. JAH conceived the study, secured funding, contributed to its design and coordination, and participated in interpretation of the data and extensive preparation of the final manuscript. All authors read and approved the final manuscript.
